# Antagonist muscle activity during reactive balance responses is elevated in Parkinson’s disease and in balance impairment

**DOI:** 10.1371/journal.pone.0211137

**Published:** 2019-01-25

**Authors:** Kimberly C. Lang, Madeleine E. Hackney, Lena H. Ting, J. Lucas McKay

**Affiliations:** 1 Graduate Division of Biological and Biomedical Sciences, Emory University, Atlanta, Georgia, United States of America; 2 Department of Medicine, Division of General Medicine and Geriatrics, Emory University School of Medicine, Atlanta, Georgia, United States of America; 3 Rehabilitation R&D Center, Atlanta VA Medical Center, Atlanta, Georgia, United States of America; 4 Department of Rehabilitation Medicine, Division of Physical Therapy, Emory University School of Medicine, Atlanta, Georgia, United States of America; 5 The Wallace H. Coulter Department of Biomedical Engineering at Emory University and Georgia Tech, Atlanta, Georgia, United States of America; Karolinska Institutet, SWEDEN

## Abstract

**Background:**

Abnormal antagonist leg muscle activity could indicate increased muscle co-contraction and clarify mechanisms of balance impairments in Parkinson’s disease (PD). Prior studies in carefully selected patients showed PD patients demonstrate earlier, longer, and larger antagonist muscle activation during reactive balance responses to perturbations.

**Research question:**

Here, we tested whether antagonist leg muscle activity was abnormal in a group of PD patients who were not selected for phenotype and most of whom had volunteered for exercise-based rehabilitation.

**Methods:**

We compared antagonist activation during reactive balance responses to multidirectional support-surface translation perturbations in 31 patients with mild-moderate PD (age 68±9; H&Y 1–3; UPDRS-III 32±10) and 13 matched individuals (age 65±9). We quantified modulation of muscle activity (i.e., the ability to activate and inhibit muscles appropriately according to the perturbation direction) using modulation indices (MI) derived from minimum and maximum EMG activation levels observed across perturbation directions.

**Results:**

Antagonist leg muscle activity was abnormal in unselected PD patients compared to controls. Linear mixed models identified significant associations between impaired modulation and PD (P<0.05) and PD severity (P<0.01); models assessing the entire sample without referencing PD status identified associations with balance ability (P<0.05), but not age (P = 0.10).

**Significance:**

Antagonist activity is increased during reactive balance responses in PD patients who are not selected on phenotype and are candidates for exercise-based rehabilitation. This activity may be a mechanism of balance impairment in PD and a potential rehabilitation target or outcome measure.

## Introduction

Abnormal antagonist muscle activity can cause joint stiffening by concurrently activating paired agonist and antagonist muscles (“co-contraction” or “co-activation”) [1–4], which may contribute to balance impairment in people with Parkinson’s disease (PD). Prior studies in PD patients carefully selected for postural difficulties and minimal tremor [5, 6] demonstrate earlier, longer, and larger antagonist muscle activation during reactive balance responses to perturbations of the support surface compared to controls [5–8]. Evaluation of antagonist muscle activation during balance could therefore potentially inform improved rehabilitative outcome measures (e.g., [9]). However, it is unclear whether antagonist muscle activity during reactive balance responses is abnormal in individuals with PD who are not selected by phenotype and are candidates for exercise-based rehabilitation. These individuals are often earlier in the disease course and may have milder symptoms than patients included in research studies without a rehabilitative component.

While co-contraction is a phenomenon that is not PD-specific [10], its elevation with age and PD and its effects on functional balance make it relevant to understanding balance impairment in PD. In adults without PD, muscle co-contraction is associated with functional changes in behavior, including increased sway [11–14], increased risk of falls [15, 16], and decreased functional reach distance and functional stability boundaries [13].

Here, our objective was to determine whether muscle modulation during balance responses was increased across leg muscles in people with PD who were not selected by phenotype. To the author’s knowledge, this is the first study to examine balance antagonist activity in the tremor-dominant PD phenotype. We used baseline measures from an exercise-based rehabilitation cohort study in PD patients and matched control individuals to test whether PD was associated with decreased muscle modulation. We recorded automatic postural responses induced by multidirectional translational support surface perturbations and examined subsequent muscle activation in a wider range of perturbation directions and muscles than previously reported [17, 18]. To do this, we used two adaptations of an existing modulation formula to yield individual muscles’ modulation across perturbation directions. To clarify the role of other predictors, we also performed secondary analyses to assess the associations between decreased muscle modulation and 1) age, 2) interaction between PD and age, 3) balance ability, 4) PD phenotype, and 5) PD severity.

## Methods

### Participants

We performed a cross-sectional observational study using baseline measures from a longitudinal study of exercise-based rehabilitation. PD patients (n = 34) and age-matched individuals without PD (“nonPD,” n = 16) were recruited from the Atlanta area from December 2013 through May 2017. Among PD patients, the majority (21/34) were enrolled into a two-arm randomized trial with dance-based exercise rehabilitation and non-contact control arms; the remaining patients and all matched individuals were allocated directly to the non-contact control arm. No screening on symptom phenotype was performed. Participants provided written consent according to protocols approved by the Institutional Review Boards of Emory University and/or the Georgia Institute of Technology.

Inclusion criteria were: age ≥ 35, vision corrected if necessary, ability to walk ≥ 10 feet with or without an assistive device, normal perception of vibration and light touch on feet, no dance class participation within the previous 6 months, demonstrated response to levodopa (PD only). Exclusion criteria were: significant musculoskeletal, cognitive, or neurological impairments other than PD as determined by the investigators.

After enrollment, participants were excluded from analysis for the following reasons: neurological diagnosis other than PD disclosed after study entry (N = 1 PD, N = 1 nonPD), non-compliance with OFF medication state (N = 1 PD), inability to complete reactive balance protocol (N = 1 PD), suspected undiagnosed cognitive impairment (N = 1 nonPD), technical difficulties in data processing (N = 1 nonPD).

### Assessment protocol

All participants were assessed according to a standardized protocol that spanned 3–4 hours including informed consent, collection of clinical and demographic information, and assessment of clinical and reactive balance. PD symptom severity was assessed by the Unified Parkinson’s Disease Rating Scale III (UPDRS-III, [19]), by a Movement Disorders Society-certified rater either in-person or on video (MEH). PD phenotype (tremor dominant, TD; indeterminate, ID; postural instability and gait difficulty, PIGD) was calculated using standard formulae [20]. Balance ability was assessed with the Fullerton Advanced Balance Scale (FAB [21, 22]). Freezing of gait was assessed with the Freezing of Gait Questionnaire B (FOGQ-B) [23]. Self-assessed balance confidence was assessed with the Activities-Specific Balance Confidence Scale (ABC) [24]. All PD patients were assessed in the 12-hour OFF medication state.

### Reactive balance assessments

**Fig 1:**
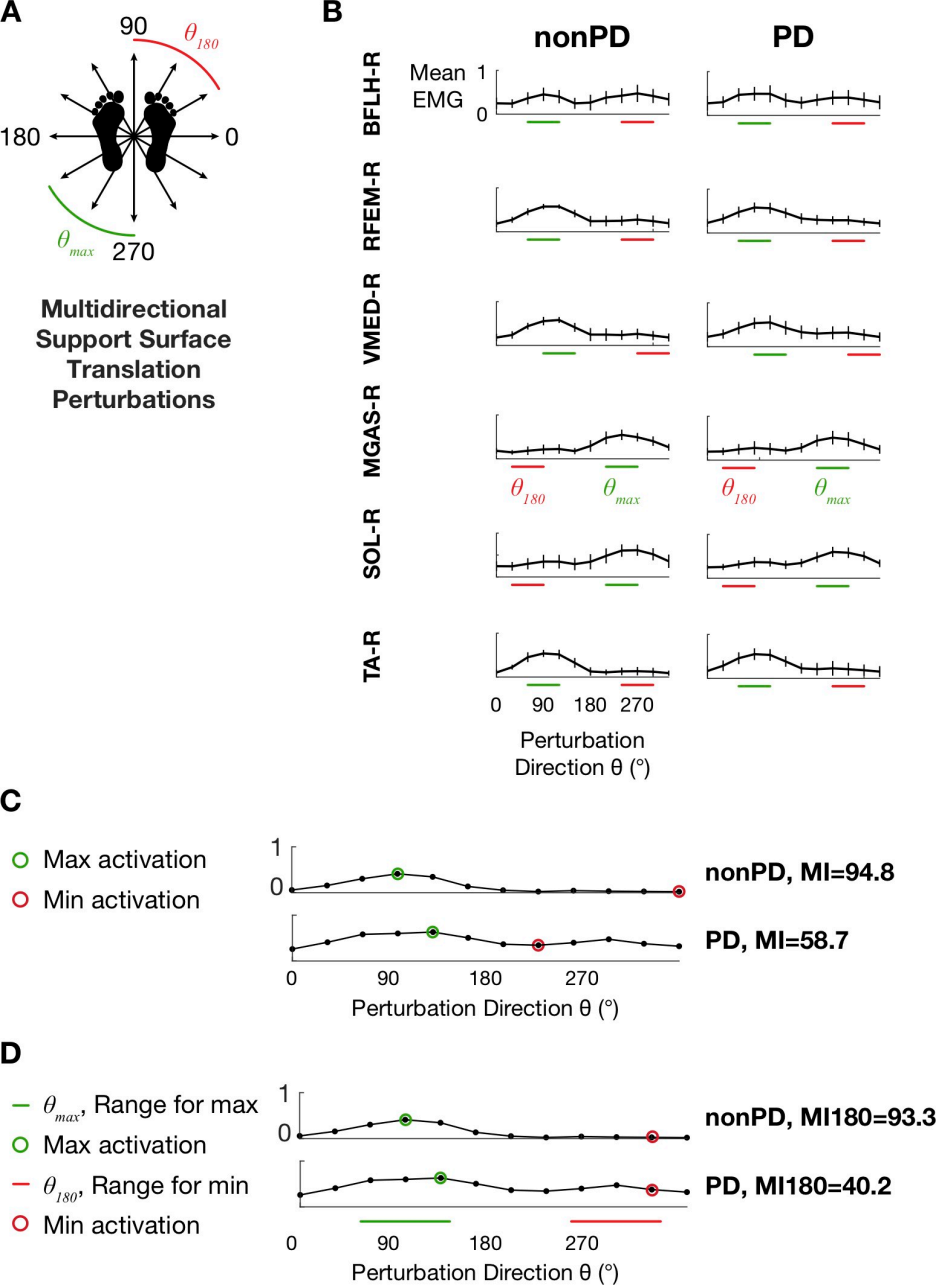
Translation perturbation paradigm and EMG tuning curves. A: Schematic depiction of multidirectional support surface translation perturbations. Green and red perturbation directions correspond to those for which maximum values were observed most frequently for MGAS-R and those directly opposite (see D). B: Tuning curves from the nonPD and PD groups depicting mean EMG activity during the APRX time bin (70–450 ms after perturbation onset). Horizontal bars indicate perturbation direction ranges *θ*_*max*_ and *θ*_180_ used for calculation of modulation index MI180. C. Examples of calculation of MI (Eq 1) and MI180 (Eq 2) for TA from two different participants.

Participants stood on a custom perturbation platform that produced ramp-and-hold support-surface translations (7.5 cm peak displacement, 15 cm/s peak velocity, 0.1 g peak acceleration) as in a previous study [9]. Feet were positioned parallel with medial aspects 28 cm apart and arms were crossed across the chest. Participants experienced 3 forward perturbations of the support surface to reduce startle effects before being tested with a set of 36 randomized perturbations in 12 evenly distributed horizontal-plane directions (Fig 1). Perturbation trials elicited stepping responses were repeated at the end of the randomized block if possible.

### EMG processing

Surface EMG activity was collected from 11 lower limb muscles: bilateral *soleus* (left, SOL-L; right, SOL-R), *medial gastrocnemius* (MGAS-L, MGAS-R), *tibialis anterior* (TA-L, TA-R), *biceps femoris long head* (BFLH-L, BFLH-R), *rectus femoris* (RFEM-L, RFEM-R) and right *vastus medialis* (VMED-R). Silver/silver chloride disc electrodes were placed 2 cm apart at the motor point [25]. EMG data were recorded using telemetered EMG (Konigsburg, Pasadena, CA) and synchronized to kinematic data (120 Hz) using Vicon motion capture equipment (Oxford Metrics, Denver, CO). EMG data were recorded at either 1080 or 1200 Hz depending on the equipment version. EMG recordings were processed offline (high-pass, 35 Hz, de-mean, rectify, low-pass, 40 Hz) [9]. Trials eliciting stepping responses or spotter intervention were identified in video records and excluded from analyses. Trials with significant EMG motion artifacts were identified by visual inspection and excluded from analyses. After exclusions due to steps or EMG quality concerns, the number of trials available per perturbation direction per participant ranged from 0 to 5 with an average of 3.0 ± 0.3.

### Muscle activity modulation indices: MI and MI180

In order to assess modulation of muscle activity during reactive balance, we computed a muscle “modulation index” that described the ability to activate and inhibit each muscle appropriately according to the perturbation direction. Because of the increased number of experimental conditions compared to previous studies, we developed two extensions of an existing modulation index that was initially developed to assess antagonist activity in only two movement directions [18]. Unlike previous work–in which the movement directions requiring each muscle to be activated (as an agonist) or inhibited (as an antagonist) were obvious from the biomechanical constraints of the task–in the multidirectional perturbation protocol used here each muscle exhibits a continuum of activity from agonist to antagonist as a function of perturbation direction.

Therefore, we calculated mean EMG levels during two time bins within each trial that encompassed the medium- and medium- and long-latency automatic postural response: 100–175 ms (APR1) and 70–450 ms (APRX) after perturbation onset [5], and subsequently assembled mean APR1 and APRX EMG levels into tuning curves describing muscle activity as a function of perturbation direction (Fig 1). Then, we used the maximum and minimum values of each tuning curve for each muscle for each participant to compute the modulation index (MI) using the following equation:

$$MI=100\cdot \frac{\max(\bar{EMG}(\theta ))-\min(\bar{EMG}(\theta ))}{\max(\bar{EMG}(\theta ))}$$ (1)

where $\bar{EMG}(\theta )$ indicates the vector of 12 mean EMG values for the 12 perturbation directions. Perturbation directions corresponding to maximum and minimum activity were identified separately for each muscle.

While the MI value reflects the greatest amount of modulation across the 12 perturbation directions, in some cases and particularly among PD patients, it did not capture abnormally elevated activity 180° from the perturbation direction for which the muscles were maximally activated, and in which the muscles could reasonably assumed to be antagonists due to the biomechanical constraints of the task. Therefore, we developed a similar formula to calculate a more physiologically-relevant index (MI180), in which the maximum value of each tuning curve was identified within the range *θ*_*max*_ of the 3 perturbation directions for which maximum EMG values were observed most frequently (Fig 1) and the minimum value was identified within the range *θ*_180_ directly opposite *θ*_*max*_:

$$MI180=100\cdot \frac{\max(\bar{EMG}(\theta_{max}))-\min(\bar{EMG}(\theta_{180}))}{\max(\bar{EMG}(\theta_{max}))}$$ (2)

Where $\bar{EMG}(\theta_{max})$ indicates the vector of 3 mean EMG values for the 3 perturbation directions included in *θ*_*max*_, and $\bar{EMG}(\theta_{180})$ corresponds similarly to the vector of 3 mean EMG values for *θ*_180_.

### Statistical analysis

Differences between the PD and nonPD groups in demographic and clinical variables were assessed with chi-squared tests and independent samples *t*-tests as appropriate.

For each muscle recorded, separate chi-squared tests of homogeneity were performed to assess crude differences in modulation between participants with vs. without PD, between participants above vs. below the sample median in age, and between participants above vs. below the sample median in balance ability, as assessed by FAB. For these tests, modulation indices (MI and MI180 in both APR1 and APRX) were dichotomized about median values. Muscles from the left and right leg of each participant were assumed to be independent observations for these tests. Associations between predictors (PD, age above the sample median, and balance ability below the sample median) and the presence of MI below the median were expressed as odds ratios (OR) ±95% CI). OR>1 indicate strong associations between the presence of a given predictor and the presence of low modulation. Primary analyses were conducted with MI in APRX (detailed below) and repeated with MI in APR1 and MI180 in both APR1 and APRX.

In order to estimate the overall association between study variables and overall modulation across muscles, multivariate linear regression analyses were then used to further examine the effects of predictors of interest, including PD, age, balance, PD severity, PD phenotype, and the interaction between PD and age.

To test whether the presence of PD was associated with muscle modulation, we fit the following linear mixed model:

$${MI}_{ijk}=\beta_{0}+\beta_{PD}\cdot PD+\sum_{i=1}^{N_{m}-1}\beta_{1i}\cdot {Muscle}_{i}+\sum_{j=1}^{N_{p}-1}\beta_{2j}\cdot {Participant}_{j}+\epsilon_{ijk}$$ (3)

in order to evaluate the following null hypothesis with an *F* test:

$$H_{01}:\beta_{PD}=0$$ (4)

In Eq 3, the indicator variable *PD* is 1 for participants with PD and 0 otherwise, *β*_1*i*_ is the beta coefficient for the fixed effect of muscle *i* (with TA as the reference group) and *β*_2*j*_ is the beta coefficient for the random effect of participant *j*.

To test whether age was associated with muscle modulation, we fit the following model:

$${MI}_{ijk}=\beta_{0}+\beta_{Age}\cdot {Age}_{c}+\sum_{i=1}^{N_{m}-1}\beta_{1i}\cdot {Muscle}_{i}+\sum_{j=1}^{N_{p}-1}\beta_{2j}\cdot {Participant}_{j}+\epsilon_{ijk}$$ (5)

where *Age*_*c*_ designates participant age centered about the sample median, and evaluated the null hypothesis:

$$H_{02}:\beta_{Age}=0$$ (6)

Similarly, to test whether balance ability as measured by FAB was associated with muscle modulation, we fit the following linear mixed model:

$${MI}_{ijk}=\beta_{0}+\beta_{FAB}\cdot FAB+\sum_{i=1}^{N_{m}-1}\beta_{1i}\cdot {Muscle}_{i}+\sum_{j=1}^{N_{p}-1}\beta_{2j}\cdot {Participant}_{j}+\beta_{3j}\cdot {Age}_{c}+\epsilon_{ijk}$$ (7)

Where *FAB* designates total FAB score, and the following null hypothesis was evaluated with a F-test:

$$H_{03}:\beta_{FAB}=0$$ (8)

Additional linear mixed models evaluating associations between additional candidate predictor variables and modulation are presented in S1 File.

## Results

### Participant characteristics

Demographic and clinical characteristics of the study participants are presented in Table 1. No significant differences were observed between the PD and nonPD groups in sex, age, or BMI. Compared to nonPD, the PD group had significantly poorer balance performance on FAB, BBS, and DGI (all P values<0.01), significantly increased prevalence of previous falls (P = 0.03), and significantly decreased self-assessed balance confidence (P<0.01).

**Table 1:** Demographic and clinical characteristics of study participants with and without Parkinson’s disease (PD).

	PD (N = 31)	nonPD (N = 13)	P Value
Sex (N, %)			0.60
Male	17, 55%	6, 46%	
Female	14, 45%	7, 54%	
Age, y, mean±SD	67.6 ± 8.8	64.5 ± 8.8	0.28
BMI, kg/m^2^, mean±SD	25.6 ± 4.0	26.0 ± 3.8	0.76
Behavioral balance measures			
BBS (0–56), mean±SD	52.2 ± 4.4	55.1 ± 1.3	<0.01*
FAB (0–40), mean±SD	29.2 ± 5.7	33.1 ± 3.1	<0.01*
DGI (0–24), mean±SD^a^	20.0 ± 3.5	22.5 ± 1.3	<0.01*
Balance Confidence, ABC (0–100%), mean±SD^b^	86.1±11.7	96.6±3.1	<0.01*
Fall History			0.03*
0 falls in previous 12 months, (N, %)	13, 42%	10, 77%	
≥1 fall in previous 12 months, (N, %)	18, 58%	3, 23%	
PD clinical features			
PD duration, y, mean±SD	7.5 ± 5.9	-	
UPDRS-III (0–108), mean±SD	31.7 ± 9.5	-	
UPDRS items, mean±SD		-	
Leg rigidity (III.22, 0–8)	1.9 ± 2.0	-	
Posture (III.28, 0–4)	1.0 ± 1.0	-	
Gait (III.29, 0–4)	1.1 ± 0.6	-	
Postural stability (III.30, 0–4)	0.8 ± 0.7	-	
Modified Hoehn & Yahr Stage, (N, %)		-	
1	1, 3%	-	
1.5	5, 16%	-	
2	13, 42%	-	
2.5	4, 13%	-	
3	8, 26%	-	
PD phenotype, (N, %)		-	
Postural Instability and Gait Disability (PIGD)	19, 61%	-	
Indeterminate (ID)	3, 10%	-	
Tremor-Dominant (TD)	9, 29%	-	
Freezing of Gait, (N, %)^a^		-	
Freezer	14, 45%	-	
Non-freezer	15, 48%	-	

Abbreviations: BBS, Berg Balance Scale; FAB, Fullerton Advanced Balance Scale; DGI, Dynamic Gait Index; ABC, Activities-Specific Balance Confidence Scale.

^a^PD N = 29.

^b^PD N = 28, nonPD N = 12.

*P<0.05.

### Description of muscle activity across perturbation directions

Tuning curves exhibited clear cosine tuning (Fig 1) consistent with those reported previously in the literature [5, 26]. Average APRX tuning curve widths at half maximum [27] were 115±7° and 111±11° for the nonPD and PD groups, respectively. Across all subjects and muscles, mean values of modulation indices in APRX were 71.9±12.9, 36.4–96.6 [mean±SD, range] for MI and 59.4±31.8, -301.6–94.8 for MI180. In APR1, the mean values were 70.8±15.2, 20.7–98.4 (MI) and 63.1±24.2, -160.3–98.4 (MI180). Negative values observed in MI180 corresponded to tuning curves in which muscles were more strongly activated in the *θ*_*180*_ range of perturbation directions and accounted for a small percentage of tuning curves in both the PD (2.4% in APRX, 1.2% in APR1) and nonPD groups (3.5% in APRX, 0.7% in APR1).

### PD, age, and impaired balance ability were associated with impaired modulation in some individual muscles in univariate analyses

**Fig 2:**
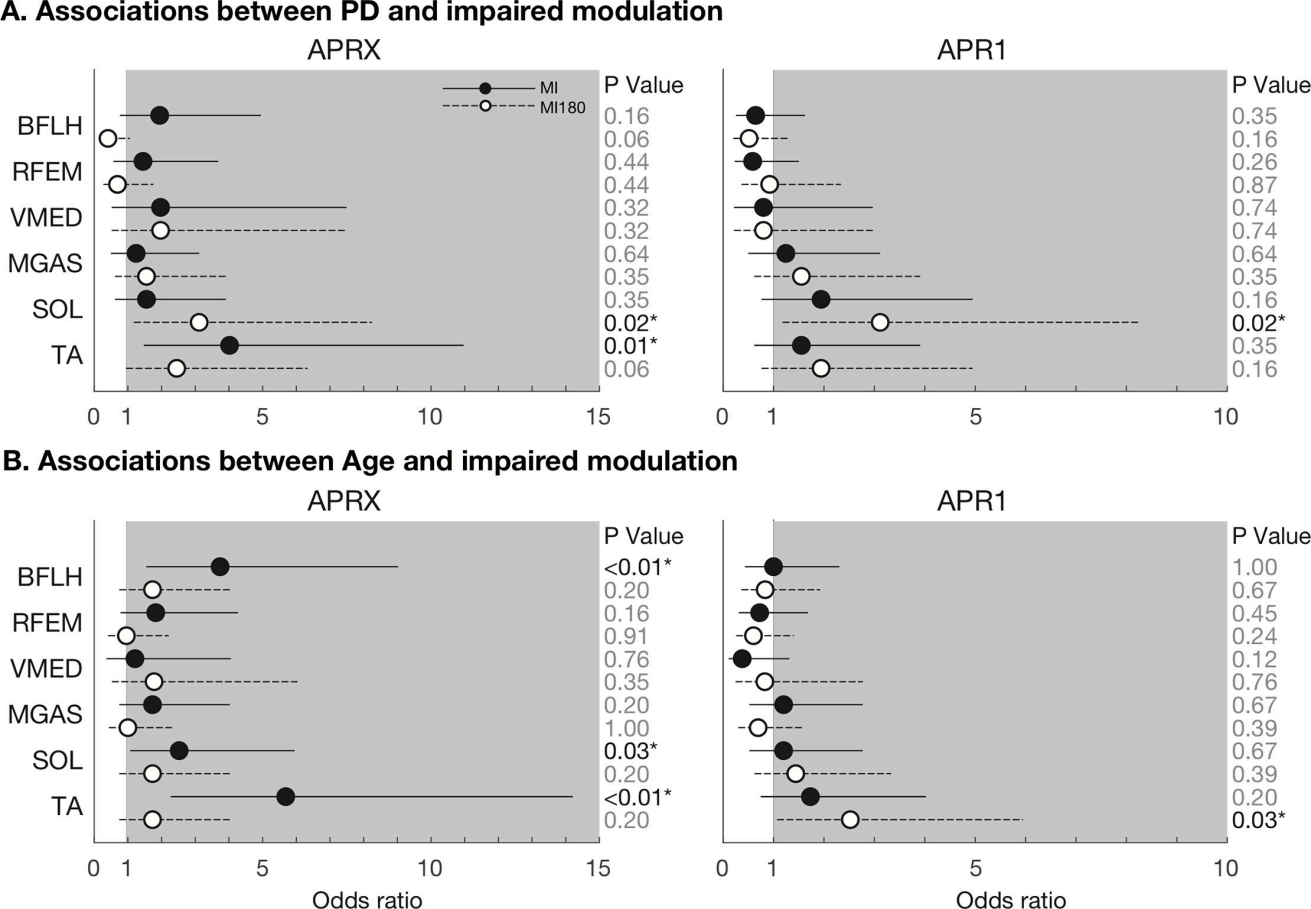
**Associations between PD (A) and Age (B) and impaired modulation in analyses of individual muscles.** Associations are described as Odds Ratios (OR) calculated separately using both MI and MI180 modulation indices derived from both APR1 and APRX time bins. Solid lines and dots represent the OR and 95% confidence limits for modulation index MI; dashed lines and open dots represent modulation index MI180. Odds ratios > 1 (shaded area) indicate that the presence of the risk factor (PD or Age) is strongly associated with the presence of impaired modulation for that muscle.

Univariate analyses showed that PD was associated with lower MI for each muscle analyzed during the APRX time window (Fig 2A, filled circles; note OR >1). This association was statistically significant for TA (OR = 4.02, P = 0.01). PD was associated with lower MI180 in 4/6 muscles analyzed during APRX (Fig 2A, unfilled circles). Age was also associated with lower MI in APRX (OR: 2.79±1.67, range 1.21–5.69), particularly for BFLH (P<0.01), SOL (P<0.05), and TA (P<0.001) (Fig 2B). Low FAB score was associated with lower MI for both BFLH (OR 2.52, 95% CL: 1.07–5.95, P = 0.03) and TA (OR: 4.59, 95% CL: 1.87–11.26, P<0.01) during APRX. Analyses during APR1 showed inconsistent associations between PD and impaired modulation (significant in SOL (OR: 3.12 [1.18–8.25], P = 0.02); Fig 2A).

Across muscles, linear mixed models identified significant associations between PD (P<0.05) and PD severity (P<0.01) and decreased MI during APRX (Table 2). Higher FAB score was also significantly associated with increased MI during APRX (P = 0.047). There was only marginal evidence of an association between increased age and decreased MI in the sample (P = 0.10), or, similarly, for interaction between PD and age in the effect on MI (P = 0.13). Linear mixed models that stratified the PD group according to PD phenotype identified strong associations between each phenotype (TD, ID, and PIGD) and decreased MI although identified parameters were only marginally significant (P = 0.06, 0.05, 0.15). Associations between these predictor variables and MI180 were the same in direction but decreased in magnitude by ≈34%. The only exception to this was that no association was identified between FAB score and MI180. No significant associations between predictors and modulation indices were identified in analyses of APR1 (S1 File).

**Table 2:** Associations between predictors of interest and muscle modulation indices MI and MI180.

		MI			MI180	
Predictor	*β*	95% CI	P Value	*β*	95% CI	P Value
PD	-4.26	-8.31, -0.21	0.04*	-3.34	-11.66, 4.98	0.43
Age	-0.18	-0.40, 0.03	0.10	-0.14	-0.58, 0.30	0.53
FAB	0.38	0.005, 0.75	<0.05*	-0.04	-0.83, 0.74	0.91
PD Severity	-0.16	-0.26, -0.05	<0.01*	-0.06	-0.29, 0.18	0.64
PD Phenotype						
PIGD	-3.25	-7.67, 1.18	0.15	-2.41	-11.64, 6.82	0.61
TD	-5.22	-10.55, 0.12	0.06	-3.85	-15.00, 7.29	0.50
ID	-7.82	-15.69, 0.04	0.05	-7.70	-24.11, 8.71	0.36
PD•Age	-0.36	-0.83, 0.10	0.13	-0.09	-1.09, 0.91	0.86

*P<0.05. Abbreviations: FAB, Fullerton Advanced Balance Scale; PIGD, Postural Instability and Gait Difficulty; TD, Tremor-Dominant; ID, Indeterminate. Mixed model results reflect the APRX time window.

## Discussion

The main result of this study was that leg muscle activity during reactive balance was abnormal in a group of mild-moderate PD patients who were unselected for phenotype. We found that lower muscle modulation across perturbation directions–an estimate of an impaired ability to appropriately inhibit muscles according to the biomechanical requirements of the balance task– was predicted by the presence of PD and by PD severity, and that, importantly, these findings were common across the TD, PIGD, and indeterminate phenotypes. Overall, the current results are consistent with the findings of previous studies in PD patients [5, 7], many of which were conducted before standardized methods of calculating and reporting PD phenotypes based on UPDRS items were in common use, which supports the generalizability of earlier results. Taken together, these results provide additional evidence that antagonist muscle activation could be a useful rehabilitative target.

Our main motivation for doing this study was to test whether abnormal reactive balance muscle activity identified in PD patients selected for “gait and postural abnormalities” [6] or “axial and/or postural problems and minimal tremor” [5], would also be present among PD patients representative of those interested in rehabilitation. While carefully selecting patients is clearly appropriate from a foundational research perspective as it decreases variability, we propose that it is critical to establish that the results generalize to rehabilitation and other therapeutic contexts in which it is typically impractical and uncommon to restrict enrollment to certain subgroups of patients. Notably, we found that the patients in our sample (average PD duration 7.5±5.9 y) were substantially earlier in the disease course than those in previous studies [5–8], in which the average PD duration was 12.5±7.3 y.

Importantly, while we anticipated that we would see differences between the PIGD and TD phenotypes on the balance task here, we found that all phenotypes were generally associated with impaired muscle modulation. Because earlier studies [5, 7] had selected for patients with significant postural signs, we reasoned that they largely were reporting on patients who would now be classified as PIGD. Therefore, we expected that the strongest associations with impaired modulation would be observed among the PIGD group, with null associations among TD. However, that was not borne out in the data. Identified regression coefficients were the same direction and of similar magnitude (-3.3, -5.2, and -7.8 for PIGD, TD, and Indeterminate) to the overall coefficient for PD (-4.3). Although these were not statistically significant–and therefore, we cannot rule out that these associations occurred due to chance–this model provides evidence that the association between PD and muscle modulation probably does not vary qualitatively across phenotypes. One potential explanation for the fact that the strongest associations were observed among the Indeterminate group is that these individuals may have had the most severe overall symptoms, with tremor, bradykinesia, and posture/gait problems all represented; however, we did not have the sample size to test this quantitatively.

While it is difficult to compare our nonPD group to those of previous studies–there are no obvious clinical variables to use–it is encouraging that the prevalence of previous falls in our nonPD group recruited from the metro Atlanta area (28%) was very similar to that reported among the spouses of PD patients in a longitudinal study conducted in the Netherlands (27%, [28]). This provides some evidence that the neurotypical nonPD group here is overall comparable to those recruited from other geographic regions (i.e., Washington and Oregon [5, 6, 8], Western Europe [7]) with different sociodemographic profiles.

One important limitation to note is that although we examined a larger sample of patients (n = 31) than many studies (n = 9–13 patients, [5–7, 9]), due to sample size limitations we were unable to impose the most stringent criteria for phenotype classification that are currently recommended [29]. Therefore it remains to be seen whether the associations between phenotypes and modulation reported here would be affected by the use of more stringent criteria, although based on the strong associations with impaired modulation observed in all phenotype groups we believe it to be unlikely. We also note that the separate mixed models approach used here was designed to balance interpretability and statistical precision in a modest sample (N = 44); however, in a larger study this could be accomplished with improved precision with a single model with appropriate interaction terms.

We were surprised that age was not significantly associated with muscle modulation here (P = 0.10), given that co-contraction is elevated in neurotypical older adults compared to young adults [10]. Although we did not observe a strong overall effect of age on muscle modulation in this sample, which ranged from 39–86, we speculate that including young adult participants would probably have resulted in a clear (although likely nonlinear) effect of age, based on the substantial work in the literature demonstrating increases in co-contraction and other changes in older adults vs. young adults–but not necessarily vs. middle-aged adults [1–4]. We noted age effects in analyses of the most proximal and most distal muscles examined. One potential explanation for this is that aging may disrupt the distal-to-proximal temporal organization of muscle responses [30], and that if younger patients were included an overall effect of age across muscles would have been identified.

The presence of a significant association between FAB score and muscle modulation supports the idea that outcome measures derived from antagonist muscle activation could be useful in the general geriatric population, although more studies are required to confirm this. Reports suggest that training may reduce co-contraction during postural control in neurotypical older adults [31] and PD [9]. However, in the linear mixed model used here (Eq 7) sample size prevented us from controlling for the presence of PD. The identified association may in part reflect a PD effect rather than a balance effect *per se*–in particular given that the PD group performed significantly more poorly on FAB (P<0.01; Table 1). Analyses here also used a median split dichotomization for FAB; although currently unavailable for this relatively new instrument, analyses using cut-off values for fall risk among PD could improve the precision and interpretation of these results. Finally, we note that due to the substantially decreased balance confidence and increased prevalence of fall history in the PD group, it is possible that associations between PD and impaired modulation reflect the presence of fall history rather than PD primary disease processes. Studies in early patients are required to evaluate this.

From a methodological perspective, the adapted modulation indices developed here may be useful in contexts other than reactive balance for capturing abnormal antagonist muscle modulation without requiring pre-specified directions of agonist and antagonist activity. These results offer a measure of the greatest possible amount of modulation (MI) and a measure of the amount of modulation that occurs when effective agonist activity is important for reactive balance (MI180). MI180 also captures instances of antagonist activity that are greater than agonist activity, which were infrequent here (<3% of muscles). In post hoc exploratory analyses, we found that in some cases PD patients performed well on MI and FAB in spite of low values on MI180 (S1 File). Based on this, we speculate that PD patients with elevated antagonist activity– as measured by MI180 – might compensate with more finely tuned agonist activity–as measured by MI–in order to perform higher overall on FAB. Of the two indices, we consider MI more general for capturing antagonistic activity, as it imposes fewer constraints on the underlying EMG data. Importantly, the modulation indices developed here do not depend on the use of fixed time windows, which were used here for simplicity. Both indices could be modified to use time windows identified with automated processes (e.g., [32]) to increase precision. We speculate that the use of variable time windows would have improved the ability to identify impaired modulation in APR1.

In summary, we found evidence that the presence of PD, PD severity, and reduced balance ability were related to a measure of elevated leg muscle antagonist activity during reactive balance. It remains to be seen whether abnormal muscle activity results from primary PD disease processes, or represents, a compensatory strategy or a potentially maladaptive compensatory strategy. However, our findings suggest that there is a relationship between antagonist activity and balance impairment in PD that generally holds for the TD and PIGD phenotypes. Consequently, elevated antagonist activity and the resulting co-contraction could be a useful rehabilitation target or outcome measure for balance rehabilitation.

## Supporting information

S1 File Supplementary material. (PDF)

S2 File Supporting data. (CSV)
