# Supplementary material for: Antagonist muscle activity during reactive balance responses is elevated in Parkinson’s disease and in balance impairment
Source: PLoS One. 2019 Jan 25;14(1):e0211137. doi: 10.1371/journal.pone.0211137 (PMC6347183; doi:10.1371/journal.pone.0211137)
Supplement: S1 File — (PDF) [file pone.0211137.s001.pdf]

Supplementary Information 1: Antagonist muscle activity during reactive balance responses is elevated in Parkinson's disease and in balance impairment.

1. Additional linear mixed models

In addition to the linear mixed models described in the main text, we fit the following linear mixed models in order to evaluate associations between additional candidate predictor variables and muscle modulation.

1.1. Interaction between PD and age

To test whether associations between PD and modulation were modified by age, we fit the following linear mixed model with an interaction term:

$$\begin{aligned} MI_{ijk} =& \beta_0 + \beta_{PD \cdot Age} \cdot PD \cdot Age_c \\ &+ \sum_{i=1}^{N_m - 1} \beta_{1i} \cdot Muscle_i \\ &+ \sum_{j=1}^{N_p - 1} \beta_{2j} \cdot Participant_j \\ &+ \beta_{3j} \cdot PD \\ &+ \beta_{4j} \cdot Age_c \\ &+ \epsilon_{ijk} \end{aligned} \quad \text{(S1)}$$

with the following null hypothesis:

$$H_{04} : \beta_{PD \cdot Age} = 0$$

1.2. PD phenotype

To test whether phenotype (TD, ID, PIGD, nonPD) was associated with MI modulation during APRX across all muscles, we fit the following linear mixed model, with variables as defined in the main text:

$$\begin{aligned} MI_{ijk} = & \beta_0 + \sum_{l=1}^{N_{Pheno}-1} \beta_{Pheno} \cdot Pheno \\ & + \sum_{i=1}^{N_m-1} \beta_{1i} \cdot Muscle_i \\ & + \sum_{j=1}^{N_p-1} \beta_{2j} \cdot Participant_j \\ & + \epsilon_{ijk} \end{aligned} \quad \text{(S2)}$$

where β_{Pheno} refers to the beta coefficient for phenotype I, with nonPD as the reference group.

The following null hypothesis was evaluated with a Type III F-test:

$$H_{05} : \beta_{Pheno} = 0$$

1.3. PD severity

To test whether PD severity (UPDRS-III score) was associated with MI modulation during APRX across all muscles, we fit the following linear mixed model:

$$\begin{aligned} MI_{ijk} = & \beta_0 + \beta_{PD\,Severity} \cdot UPDRSIII \\ & + \sum_{i=1}^{N_m-1} \beta_{1i} \cdot Muscle_i \\ & + \sum_{j=1}^{N_p-1} \beta_{2j} \cdot Participant_j \\ & + \epsilon_{ijk} \end{aligned} \quad \text{(S3)}$$

where $\beta_{PDSeverity}$ refers to the beta coefficient for UPDRS-III score. The following null hypothesis was evaluated with a Type III F-test:

$$H_{06} : \beta_{PD\,Severity} = 0$$

2. Associations between study variables and modulation indices in APR1.

Across muscles, linear mixed models identified no significant associations between predictors and either modulation index in the APR1 time bin (Table S1).

Table S1. Associations between predictors of interest and muscle modulation indices MI and MI180 calculated during the APR1 time window.

	MI			MI180		
Predictor	β	95% CI	P Value	β	95% CI	P Value
PD	0.31	-4.21, 4.83	0.89	-1.37	-9.35, 6.61	0.74
Age	-0.06	-0.30, 0.17	0.61	-0.02	-0.44, 0.40	0.93
FAB	0.29	-0.13, 0.71	0.17	0.31	-0.44, 1.06	0.42
PD Severity	-0.02	-0.15, 0.10	0.72	-0.01	-0.23, 0.22	0.96
PD Phenotype						
PIGD	-0.55	-5.55, 4.45	0.83	-3.48	-12.23, 5.27	0.44
TD	1.92	-4.10, 7.95	0.53	2.30	-8.25, 12.86	0.67
ID	0.99	-7.90, 9.88	0.83	1.04	-14.52, 16.60	0.90
PD•Age	-0.03	-0.58, 0.51	0.90	-0.10	-1.06, 0.86	0.84

*p<0.05. Abbreviations: FAB, Fullerton Advanced Balance Scale; PIGD, Postural Instability and Gait Difficulty; TD, Tremor-Dominant; ID, Indeterminate.

3. Associations between MI, MI180 and FAB

In primary analyses of associations between PD- or age-related factors and muscle modulation, we found overall similar associations using MI and MI180, in the sense that identified regression coefficients were of the same sign and similar approximate magnitude. Our interpretation of this is that the two indices describe deficits in muscle modulation due to PD and /or aging generally similarly, with MI being somewhat more precise because it does not prescribe 180° separation between directions of maximum and minimum activation. One notable exception to this is that a relatively strong association between high FAB and high muscle modulation was observed for MI, but not for MI180. This suggests that muscle activity in perturbation directions strictly 180° opposite from the agonist direction is not associated with FAB score.

In order to further investigate discrepancies in associations between MI and FAB and between MI180 and FAB (Table 2), we performed additional exploratory regression analyses relating MI and MI180 to FAB score in each of the PD and nonPD groups (Fig S1).

These analyses identified only one association significant at $P<0.05$, between MI and FAB among the PD group. (Note that P values shown are calculated from crude linear regressions,

are not corrected for repeated measures of individual subjects or muscles, and are therefore intended to be interpreted only as an indicator of effect size.) Inspection of plots suggested that in some cases, PD patients with poorer modulation on MI180 are nevertheless able to perform better on both MI and on FAB (Fig S1, Panel A1, Top Left, Panel A3, Bottom Right). This relationship was not observed among the nonPD group, which on inspection exhibited a tighter relationship between MI and MI180 (compare top and bottom).

Based on these results, we speculate that some PD patients may be able to compensate for abnormal antagonist activation of muscles – as evidenced by lower values of MI180 – with increased modulation of muscle activity at other directions on the muscle tuning curve – as evidence by higher values of MI – and as a result are able to perform higher on FAB.

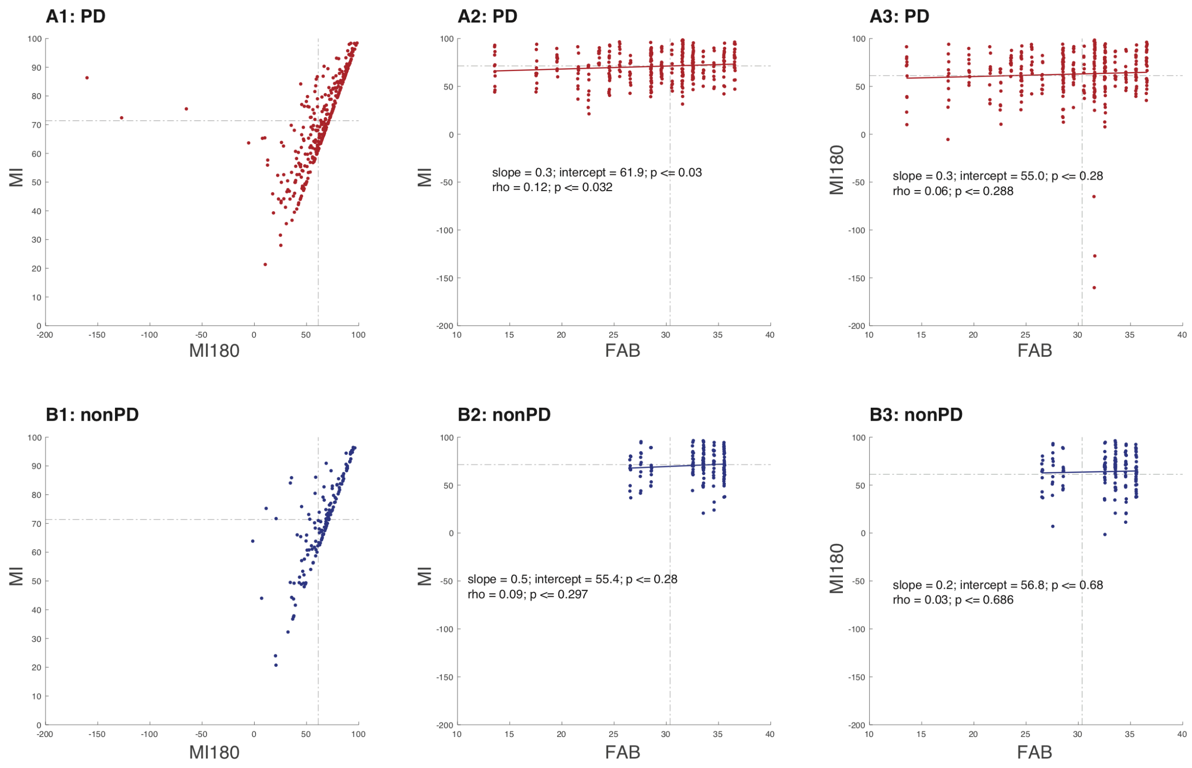

Figure S1. Results of stratified regression analyses demonstrating associations between FAB, MI, and MI180 in the nonPD and PD groups. P values shown are calculated from crude linear regressions and are not corrected for repeated measures of individual subjects or muscles. Reference lines indicate mean values. In panels A2,3 and B2,3 a small amount of horizontal jitter has been added for visibility.
